# Toward accessible MRI: SDR4MR, a simple RF pulse monitoring technique using an inexpensive software-defined radio

**DOI:** 10.1007/s10334-025-01249-z

**Published:** 2025-04-19

**Authors:** Kouame Ferdinand Kouakou, Anita Paisant, Christophe Aube, Hervé Saint-Jalmes

**Affiliations:** 1 Laboratoire HIFIH-SFR ICAT, Université d’Angers, Angers, France; 2 Laboratoire LTSI-INSERM 1099, Université de Rennes, Rennes, France; 3 SIEMENS Healthcare SAS, Courbevoie, France; 4 Département de Radiologie-CHU d’Angers, Angers, France

**Keywords:** MRI, Radiofrequency, Software-defined radio, Pulse sequence

## Abstract

**Objective:**

This study evaluated the applicability and performance of the SDR4MR method at 1.5 T and 3 T across different acquisition scenarios in a clinical environment.

**Materials and methods:**

The SDR4MR hardware consists of a broadband receiver coil connected to a software-defined radio (SDR) via optional RF attenuators. The SDR stick is plugged into the computer's USB port, which runs the SDR software and a Mathematica script to decode the RF pulse sequence. Several MRI pulse sequences were recorded: (i) a multi-echo multi-slice spin echo sequence to check the SDR4MR configuration on a well-known simple sequence; (ii) 2D and 3D sequences for which detailed information is not available in the user interface.

**Results:**

The measured RF pulse sequences have been drawn in the style of illustrations found in MRI textbooks. Sequence times and amplitudes were estimated, and sequence details not described in the MRI user interface were retrieved.

**Conclusion:**

The present study demonstrated the implementation of SDR4MR on clinical scanners. This easy-to-use configuration enables precise monitoring of RF pulse sequences. This method could be further improved by taking advantage of advances in SDR hardware and software.

## Introduction

Magnetic Resonance Imaging (MRI) is a rapidly evolving technology, marked by the continuous development of complex technological tools, both hardware and software, offered by manufacturers. These tools often function as a “black box”, making it extremely challenging to gain a comprehensive understanding of their inner workings without special agreements granting access to sequence simulators provided by the manufacturers. Ensuring accurate control of sequence parameters is essential for quantitative imaging. During the optimization of a pulse sequence, manufacturer’s software often compromises between conflicting parameters. For example, when the repetition time of the sequence is constrained, magnetization preparation pulses may be shifted without the user's knowledge leading to inaccurate quantification.

Previous methods have sought to explore these “black boxes”. The ViP method [1], derived from the extension of the ERETIC method [2] to MRI, allows exploration of the MRI receiver chain and image reconstruction algorithms. Field cameras [3] provide accurate information on radiofrequency (RF) sequences and gradients. Recently, a combination of Field Camera and ViP method has been proposed to implement a new quality assurance method for MRI [4]. However, these very useful methods require the development of complex hardware and software, requiring complex and expensive environment.

The concept of software-defined radio (SDR) [5] was to connect an RF front-end to software-based demodulation functions to provide inexpensive, versatile radios that can be modified through software. Applications to MRI date back some ten years, and have led to the development of low-cost, essentially low-field NMR spectrometers [6, 7]. In a recent study [8], a simple and cost-effective alternative was introduced to specifically solve the measurement of the RF component of MRI sequences. The SDR is implemented as a USB stick directly connected to a computer, which manages the demodulation methods, adjusts the gains, and receives the demodulated data. The main advantage of this approach, called SDR for magnetic resonance (SDR4MR) is that it is independent from MRI scanner hardware and software. Moreover, the total cost of the device is under 100€, making it a highly affordable solution with the aim of making MRI more accessible [9].

The aim of this study was to assess the SDR4MR method on clinical MRI scanners at 1.5 and 3 T, across different MRI data acquisition scenarios, from basic 2D multi-slice spin-echo to three-dimensional (3D) turbo spin-echo sequence with varying flip angles, to determine its applicability and performance in clinical environments.

## Materials and methods

**Fig. 1:**
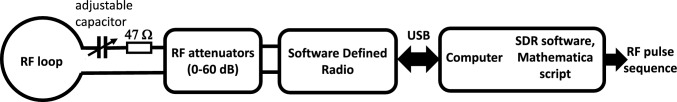
Receiving system diagram. The receiver coil is broadband to allow measurements at 1.5 T and 3 T. It is connected to the SDR via optional RF attenuators to accommodate a wide range of RF pulse amplitudes. The SDR stick is plugged into the computer's USB port, which runs the SDR software and a Mathematica script to decode the RF pulse sequence

The SDR4MR method relies on a SDR device integrating powerful hardware and a software setup to decode MRI RF pulse sequences. Figure 1 shows a schematic representation of the apparatus.

### Custom RF coil

**Fig. 2:**
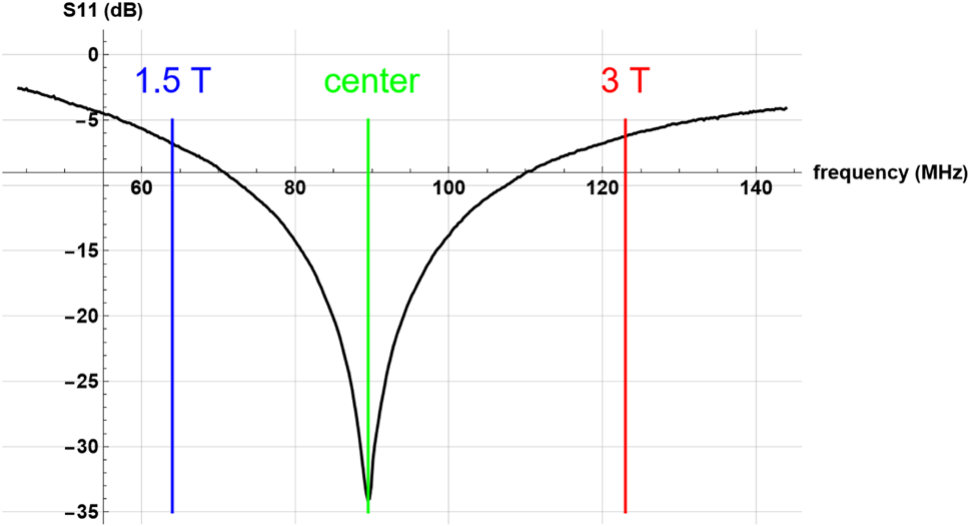
RF loop S11. The coil is tuned to 90 MHz (green) with a low-quality factor to allow RF measurements at 1.5 T (blue) and 3 T (red) without further tuning

A home-built 40 mm-diameter RF coil is tuned to a standard 50-Ω impedance, for optimizing impedance matching with the RF attenuators. The coil has a low-quality factor (1–2), which ensures a wide reception range suitable for measurements at 1.5 T and 3 T without requiring further adjustment, as shown in Fig. 2.

**Fig. 3:**
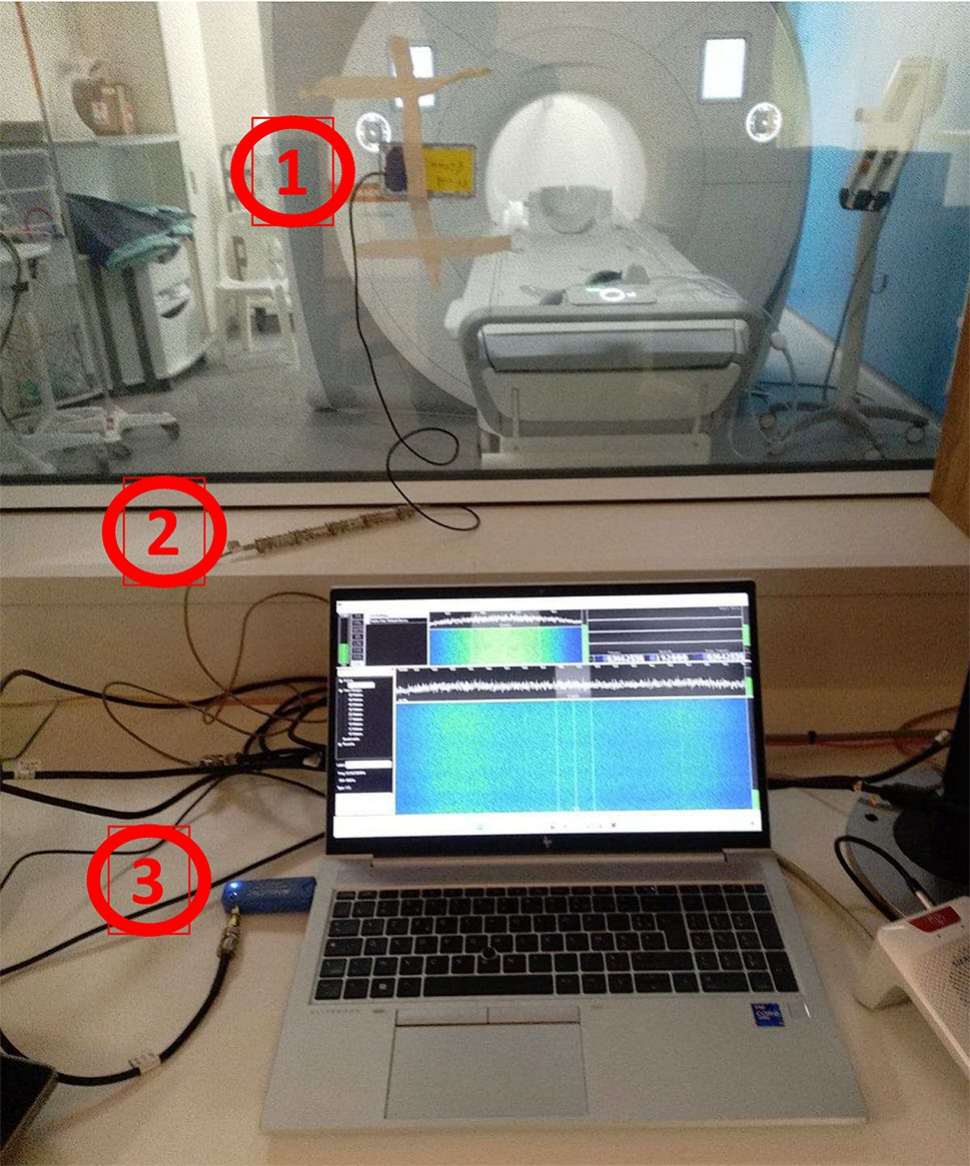
Photograph of the device. The receiver coil (1) is located inside the shielding room. Its coaxial cable passes through the waveguide and is connected, if necessary, to RF attenuators (2), before being plugged to the SDR (3) and the computer

The coil is placed outside the magnet bore, either inside or outside the shielded room. In the first case, as shown in Fig. 3, the impact of external electromagnetic interference is minimized by the RF shield, and the coil cable runs through the waveguide to the console room. The cable shield is connected to the waveguide to limit possible interference. RF attenuators (Mini-Circuits, USA) are required to reduce the powerful signal induced by the B1 RF field in the coil. Depending on the sequence and the magnetic field, attenuation of 20–40 dB was applied. In the second case, the installation is simplified by the coil placed externally against the RF shield. Depending on the effectiveness of the shielding and possible electromagnetic interference, the weak RF signal may be corrupted, limiting the available dynamic range.

### Connection to SDR and software configuration

The RF coil is connected directly or through attenuators to a commercial SDR dongle (R820T2 SDR and DVB T, NESDR Mini 2 +, NooElec, USA), a compact device that plugs into the USB port of the computer. This device combines an R820T2 tuner, an RTL2832U demodulator, and USB interface. It contains a high-precision TCXO crystal (Temperature Compensated Crystal Oscillator) with a stability of 0.5 ppm. Analog/digital conversion is performed on 8 bits. The receiving bandwidth covers 25–1750 MHz. An open-source software CubicSDR, available at cubicsdr.com, is used to control the SDR dongle and configure the reception parameters. This software was chosen from a wide range for its ability to display the demodulated signal in the time domain, enabling the RF signal of the pulse sequence to be viewed in real time and the receiver gain to be adjusted accordingly. In our case, the parameters were: 3.2 MHz sampling frequency, no direct sampling, no automatic gain control (AGC), and in-phase and quadrature (I/Q) mode activated to capture both amplitude and phase of RF signals, with an audio sampling rate of 192 kHz (the maximum), corresponding to the sampling frequency used to record the two channels I and Q in a WAV file.

The SDR is configured to operate at the magnetic resonance frequency (e.g., 63.64 MHz for 1.5 T clinical MRI and 123.6 MHz for 3 T clinical MRI in our case). The exact resonance frequency is usually available in the MRI scanner's user interface. This allows the SDR's receive frequency to be centered, and the receive bandwidth to be fully utilized for optimum RF signal measurement.

### Data acquisition and processing

The RF signals demodulated by the SDR are recorded as WAV files, at a sampling rate corresponding to the defined reception bandwidth. These files, containing raw data from the RF sequences, are subsequently analyzed using a custom Mathematica script (Wolfram Research, Inc., USA). This in-house script processes WAV files to generate temporal and frequency representations of RF pulse sequences. The script first provides a global view of the recording contained in the file, and then, the user selects parts of the file to enable precise pulse analysis in the time and frequency domains. There is absolutely no link between the MRI and the SDR. The only information used is the MRI frequency, so there is inevitably an offset between the MRI frequency and the SDR demodulation frequency. For this reason, an additional step of fine digital demodulation is performed in the Mathematica script, allowing amplitudes and phases to be recovered within a pulse. In particular, this additional demodulation function in the receive bandwidth is used to highlight frequency changes in selective pulses, for example in 2D multi-slice acquisitions. Pulse sequence information, related to both time and frequency, such as pulse duration, relative amplitudes, frequencies, and phases, is extracted. These data enable analysis of sequence characteristics, including parameters not easily accessible via the standard user interface of clinical MRI systems.

### Validation on MRI sequences

To validate the capabilities of the SDR4MR setup, several MRI pulse sequences were recorded and analyzed on a 1.5 T clinical system (Magnetom Sola, Siemens Healthineers, Erlangen, Germany) and a 3 T clinical system (Magnetom Vida, Siemens Healthineers, Erlangen, Germany). The following sequences have been selected to represent various use cases at 1.5 T:

- a multi-echo multi-slice spin-echo sequence (SEMC), five slices of 5 mm with TR/TE of 400/9.9 ms (for 1–7 echoes),
- a vendor-provided B1 mapping sequence (B1 map), with TR/TE of 4660/1.51 ms, a flip angle of 10°, a 64-matrix resolution,
- a Spin echo EPI diffusion weighted imaging (SE-EPI), five slices of 7 mm, with TR/TE = 1800 ms/48 ms, fat suppression: STIR inversion and water excitation, b = {0; 600}s/mm^2^.

These other ones were selected at 3 T:

A 3D turbo spin-echo sequence (3D SPACE) with the following common parameters: 160 slices in one slab of 165 mm, TR/TE = 1260 ms/204–206 ms, in plane 288-matrix resolution. Three variants were studied:

- with constant flip angle,
- with constant flip angle and magnetisation restoration pulse,
- with a variable flip angle optimized for a T2-weighted contrast.

The main parameters of the above sequences acquired on phantoms are summarized in Table 1.

**Table 1:** Summary of some essential parameters of the tested sequences

Name	Sequence	Variant	Preparation	TR/tr per slice/TE (ms)	Flip angle (°)	B0 (T)
SE-MC	Multi-echo (7) multi-slice (5) spin-echo			400/80/9.9	90	1.5
B1 map	Turbo flash			4660/245/1.51	10/a2	1.5
SE-EPI	Spin-echo EPI	Water excitation	STIR	1800/360/48	90	1.5
3DT2c	3D SPACE	None		1260//204	120	3
3DT2r	3D SPACE	Restoration		1260//204	120	3
3DT2v	3D SPACE	Variable angle		1260//206	120	3

The choice of the sequences was motivated by the following reasons: (i) first, to measure a multi-echo multi-slice sequence (SEMC) to check the SDR4MR configuration on a well-known simple sequence; (ii) second, to study sequences for which detailed information is not available in the user interface. The data collected from these sequences were analyzed to assess the system's ability to reproduce the characteristics of the RF pulses derived from the user interface, including durations and frequency variations between slices.

## Results

**Fig. 4:**
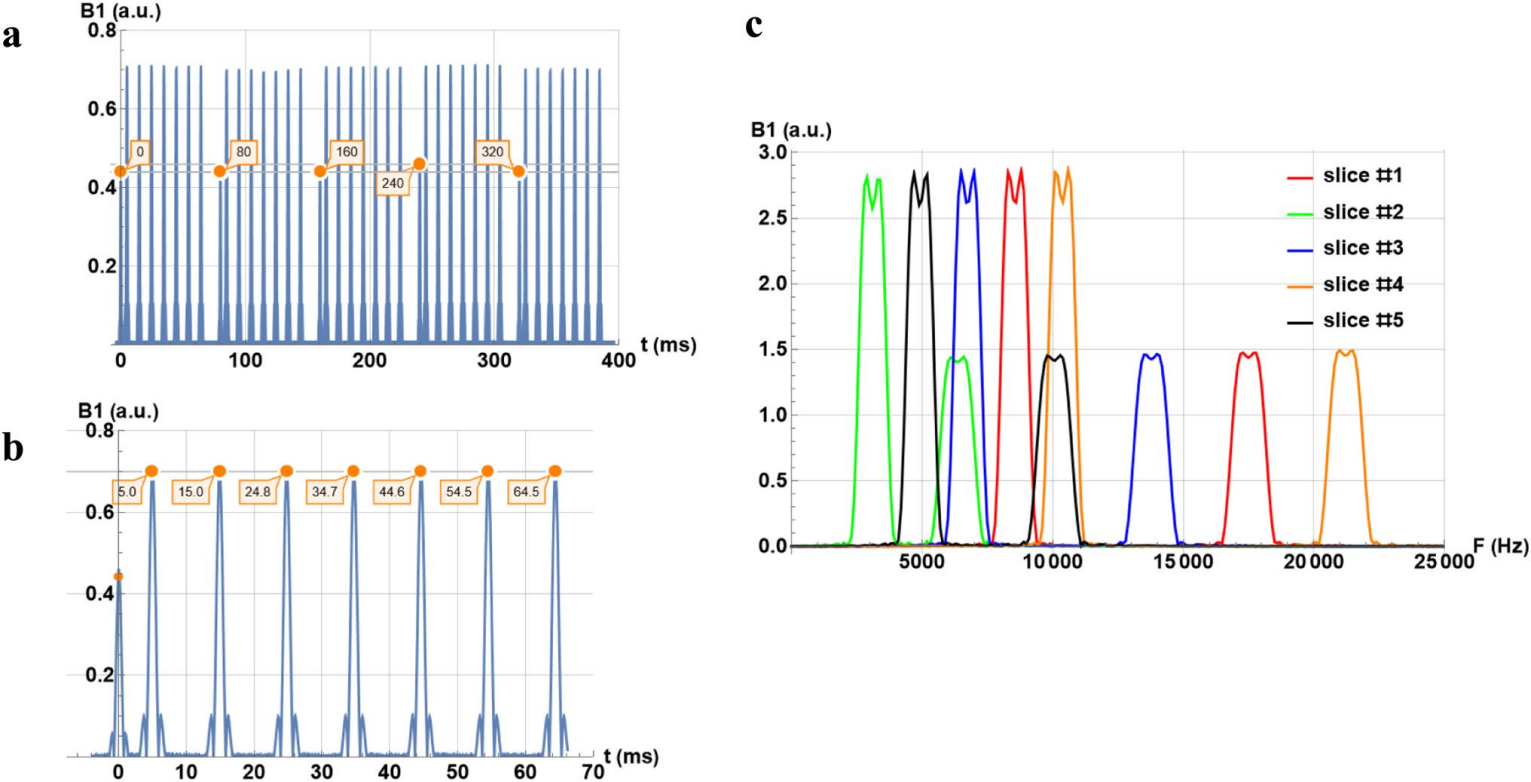
Diagrams of the SEMC pulse sequence measured by SDR4MR at 1.5 T, **a** capture of five slices of the seven-echo sequence, **b** details of the seven echoes, and **c** frequency analysis of 90° and 180° pulses for the five slices

For the SEMC sequence, Fig. 4a, b shows the recorded pulse sequence of 7 echoes and 5 slices as well as the measured timings. From these figures, the slice repetition time and echo time can be estimated at 80 ms and 10 ms, respectively, for 1 to 7 echoes. The frequencies of the 180° and 90° pulses for each of the 5 slices are represented in Fig. 4c.

**Fig. 5:**
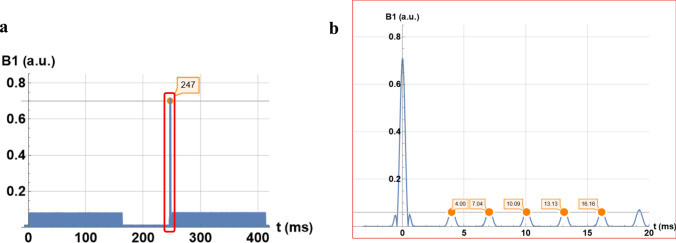
Diagrams of the B1 map pulse sequence measured by SDR4MR at 1.5 T: **a** two consecutives trains of pulses without and with the preconditioning pulse. A repetition time per slice of 247 ms is measured: **b** detail of the first pulses in red rectangle showing an echo time of 3.04 ms

A detail of the B1 map recording is shown in Fig. 5. The graph on the left shows two pulse trains of a turbo fast low-angle-shot (TurboFLASH) sequence. The last train is preceded by a sinc RF pulse. A detailed analysis of pulse occurrence resulted in a slice repetition time of 247 ms and a TurboFlash repetition time of 3.04 ms.

**Fig. 6:**
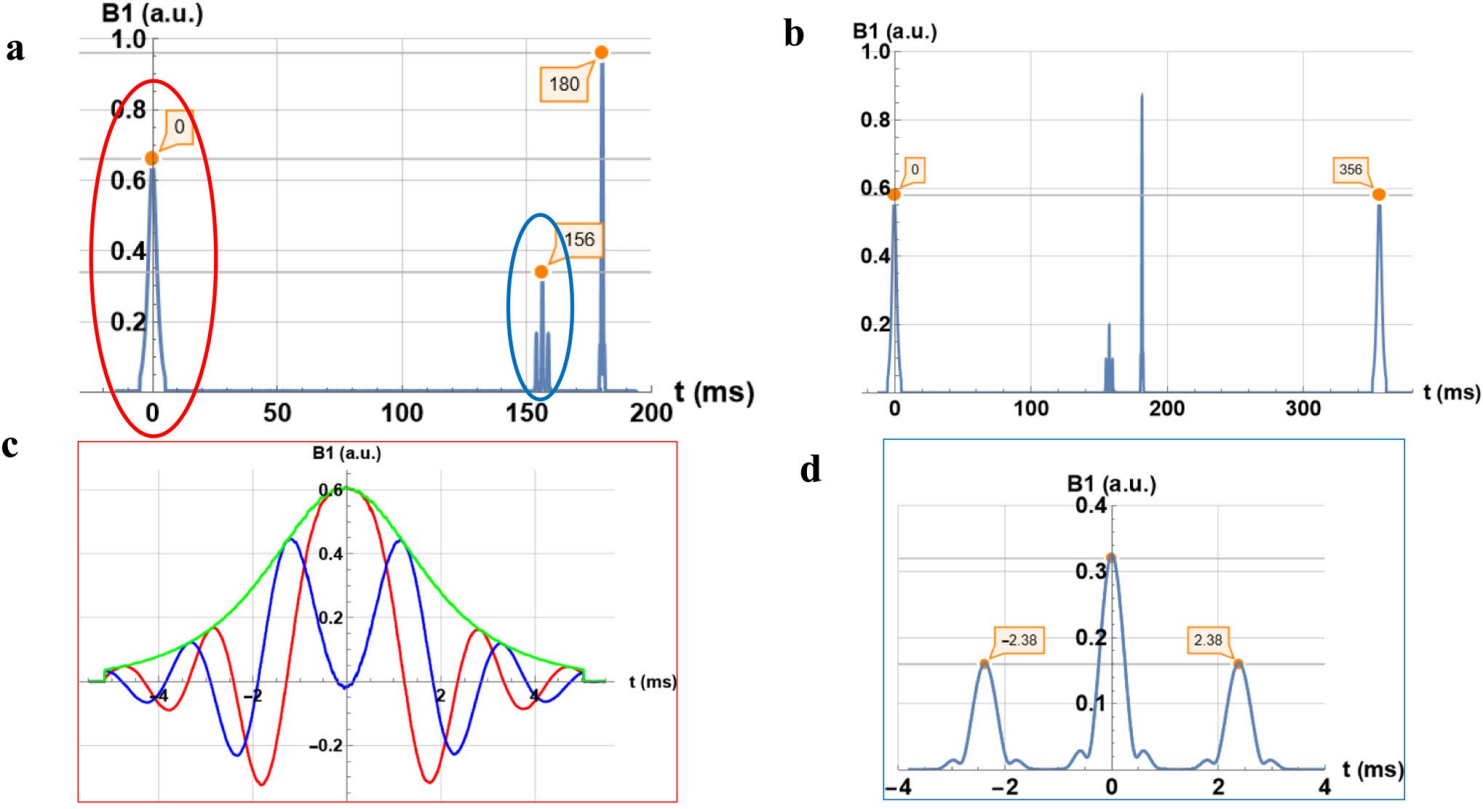
Diagrams of the diffusion pulse sequence measured by SDR4MR at 1.5 T, **a** an inversion pulse is followed by a water excitation pulse before the 180° pulse, **b** a repetition time per slice of 356 ms is measured, **c** real, imaginary parts and modulus of the inversion pulse, and **d** modulus of the water excitation pulse

The SE-EPI sequence was performed as described in Table 1 by a combination of two fat suppression techniques: a STIR inversion and a water excitation pulse. The analysis of this sequence by the SDR4MR technique is described in Fig. 6. The first graph (a) gives an overview of the RF pulse sequence. First, the sequence begins with the inversion pulse with an estimated time between this pulse and the 90° pulse of 156 ms. The measured time between the 90° pulse and the 180° pulse, which represents the half of the echo time is equal to 24 ms giving an echo time of 48 ms corresponding to the user interface value. The second graph (b) shows a repetition time per slice of 356 ms for a 360 ms prescribed value. The SDR4MR not only measures the modulus of the RF pulse, but also gives access to the real and imaginary parts. The graph (c) of Fig. 6 represents the detailed visualization of a 10 ms STIR pulse. The fourth graph (d) shows a zoom on the water excitation pulse.

**Fig. 7:**
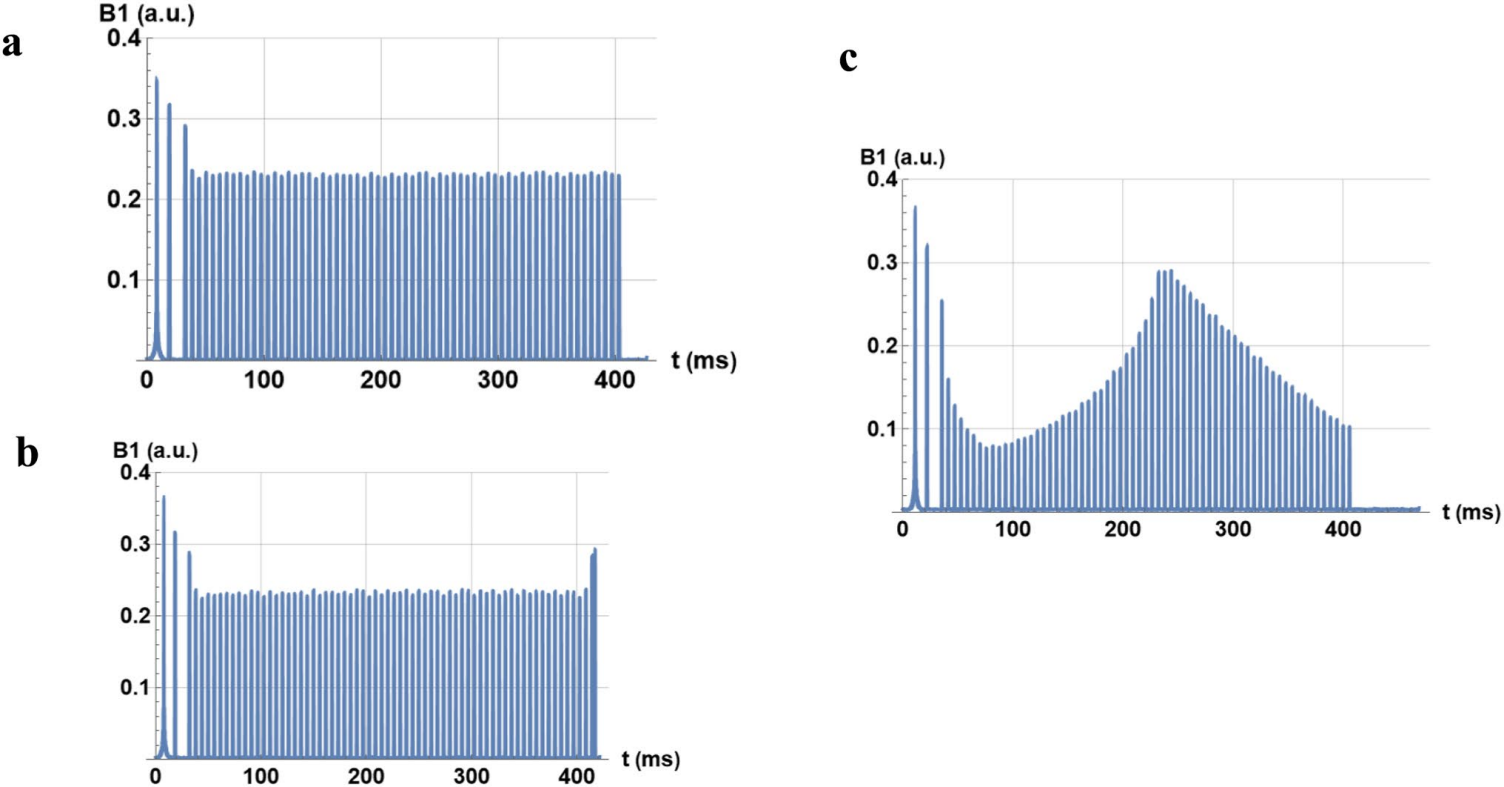
Diagrams of the 3D SPACE pulse sequences measured by SDR4MR at 3 T: **a** constant angle, **b** constant angle with restoration, and **c** variable flip angle

**Fig. 8:**
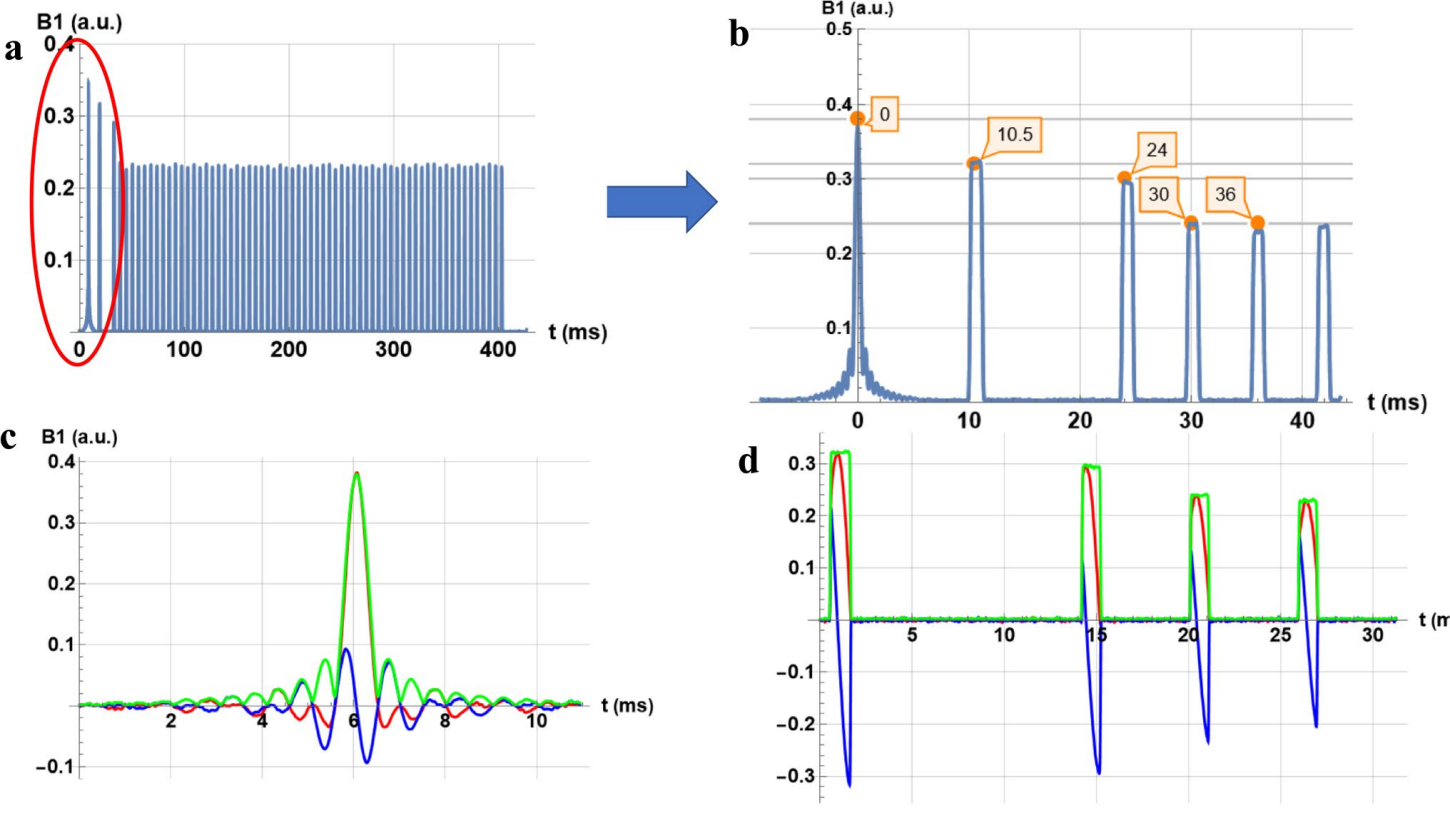
**a** 3D SPACE T2 at constant angle, **b** detail of timings of red ellipse, **c** first pulse (real red, imaginary blue, modulus, green), and **d** subsequent pulses

For the validation of the SDR4MR method at 3 T, three different variants of a 3D SPACE sequence were recorded. Figure 7 shows these variants, with a constant flip angle, respectively, without (a) and with a magnetization restoration pulse (b), and the third variant with a variable angle. The constant flip angle SPACE in Fig. 8a is enlarged to show the pulse-sequence timing diagram for the first pulses (Fig. 8b). The real, imaginary parts and the modulus of these pulses of different shapes are shown: the first (Fig. 8c) is a sinc pulse, while subsequent pulses (Fig. 8d) are rectangular.

## Discussion

In this study, we implemented a compact and inexpensive system to monitor the RF signal of pulse sequences of clinical MRI scanners at 1.5 T and 3 T. The measured parameters showed good agreement with the prescribed values when available as detailed below.

For the SEMC sequence, the measured slice repetition time of 80 ms and echo time of 10 ms agree with those prescribed on the scanner user interface to within 1% precision (Table 1). In the frequency domain (Fig. 4c), an even–odd interleaved acquisition is observed. This interleaved slice acquisition scheme is regularly used to avoid excitation leakage between slices.

The B1 map sequence is not precisely documented by the vendor. Its analysis by the SRD4MR method reveals that the turboFlash acquisition is repeated twice without and with a sinc RF preceding pulse as described by Sohae Chung et al. [10]. In addition, only the flip angle of the TurboFlash readout is available on the user interface. The angle of the preconditioning pulse was unknown. However, by taking the ratio between the amplitudes of the preconditioning pulse and the readout pulse in the frequency domain, a preconditioning pulse flip angle a2 of 80° is deduced, while a 60° value is preferred in [10].

The SE-EPI sequence analysis reveals a delay of 156 ms between the STIR pulse and the 90° pulse. This inversion time is very close to the prescribed value of 160 ms. The STIR pulse real and imaginary parts (Fig. 6c) are similar to those of a hyperbolic secant pulse often used for adiabatic inversion [11]. The water excitation scheme chosen in the user interface is not detailed. From the SDR4MR analysis, it is deduced (Fig. 6d) that it is a binomial 1–2–1 pulse with a 2.38 ms spacing between pulses in order not to affect the fat protons [12, 13].

At 3 T, the three SPACE sequences studied correspond to their description: a constant flip angle (Fig. 7a), a restoration pulse at the end of the pulse train (Fig. 7b), and a variation in flip angle of successive RF pulses (Fig. 7c). Nevertheless, the variation of the flip angle for this T2 contrast sequence seems to differ slightly from recently published patterns [14]. The interpulse delays shown in Fig. 8b are very close to those recommended by John P. Mugler III (cf. Fig. 14 of [15]).

When comparing SDR4MR time measurements with the prescribed parameters, the measurements obtained using the SDR4MR system demonstrated minimal imprecision, on the order of 1%. This error is not accurately characterized, as it is determined by measuring the time separating the maxima of two pulses (for example, 90° and 180° pulses), which is not very precise, and assuming that the parameters prescribed in the user interface are applied exactly.

Relative amplitude measurements were also consistent with expected values (cf. Fig. 4a for the 90° and 180° pulses of the spin-echo sequence, Fig. 6d for the binomial pulse). Access to both the real and imaginary component of the signal, rather than only the modulus, gives detailed view into complex RF pulses, such as adiabatic inversion (see Fig. 6c). Amplitude measurements were slightly less accurate than time measurements; this was likely due to the limited dynamic range of the SDR system.

This implementation is standalone and independent of the MRI scanner, allowing it to be used across MRI scanners of different vendors and at different field strengths. As clinical MRI scanners become increasingly complex, users have less control and understanding of the details of a pulse sequence. This SDR4MR method enables the retrieval of detailed and precise sequence information without the need for a research contract with the vendor. In the case of an existing research agreement for the development of new pulse sequences by researchers, this system can be used to verify the conformity of the pulses actually produced by the MRI scanner with the programmed sequence. This facilitates the identification of the optimal pulse sequence, which is a compromise between the desired pulse sequence with the limitation of the hardware and software (as linearity, amplitude, phase control, etc.).

Finally, this method could be used to train MRI users alongside patient scans, without slowing down the acquisition process. Additionally, it could provide service engineers with a light and simple tool to monitor the RF emission of the MRI scanner.

This system has certain limitations. To completely decode a sequence, gradient measurements are necessary, but they are beyond the scope and budget of this study. Systems such as those proposed in [3] cost several orders of magnitude more than an SDR. The RF coil measures a global signal emitted by the scanner's excitation coil, and therefore does not provide access to the individual field emitted by each element of the emission coil when several are used. The measured amplitudes are relative and not absolute, unless a precise calibration is performed in a well-defined geometrical configuration of the receiver coil, which was not the aim of this study. However, if the coil position and gain configuration remain identical, several consecutive sequences can be analyzed and compared in terms of amplitude.

The basic SDR used in this study has the advantage of being very cost-effective, but its performance is sometimes limited in low-field MRIs. The RF front-end covers a frequency range from 25 MHz to over 1 GHz, which is not suitable for low-field MRI generations. The demodulator has an 8-bit converter, which limits its dynamic range and may therefore miss subtle details of RF pulses. However, this is more than sufficient for clinical MRIs up to very high-field MRI, and the device has been successfully tested at 500 MHz [8]. For users who wish to apply it to cover the entire NMR and MRI spectrum, additional SDR units are available that cover from a few kHz (ultra-low-field) to over 1 GHz (ultra-high-field NMR). In addition, the SDR device used in this study has a dynamic range certainly limited by its ADC (~ 50 dB), although it has not been measured. On modern scanners, the B1 + maximum is quite high (> 25 uT), allowing very short hard pulses of a tenth of a ms, whereas long spectral and/or spatially selective pulses can last 10 ms or more. Considering the software-defined radio as an oscilloscope, it would be desirable to measure the amplitude of this long pulse to within a few %, which would give a rough estimate of the dynamic range required of 100 × 100 = 80 dB. It would be difficult to achieve this with an inexpensive SDR, but in the 100 € price range, dynamic ranges of 65 to 70 dB are available, which would already be much better. These SDR units with 12- to 14-bit analog-to-digital converters would also be more convenient for measuring a wide variety of RF pulses without having to modify gains or front-end attenuators.

It should also be noted that the 192 kHz reception bandwidth may sometimes pose a limitation at high-field strengths. As field strength increases, the bandwidth occupied by RF pulses also increases, particularly in 2D multi-slice experiments. This limitation applies to the basic version of CubicSDR, but several other free software packages are available to overcome this limitation.

## Conclusion

The present study demonstrated the implementation of SDR4MR on clinical scanners. This easy-to-use configuration enables precise monitoring of RF pulse sequences.

## Data Availability

All data and codes that support the findings of this study are available from the author, upon request. The current version of the Mathematica script and a sample wav file can be downloaded at https://github.com/LTSI-SDR4MR/MathematicaScript.git.
